# Lineage Divergence and Historical Gene Flow in the Chinese Horseshoe Bat (*Rhinolophus sinicus*)

**DOI:** 10.1371/journal.pone.0056786

**Published:** 2013-02-25

**Authors:** Xiuguang Mao, Guimei He, Junpeng Zhang, Stephen J. Rossiter, Shuyi Zhang

**Affiliations:** 1 Institute of Molecular Ecology and Evolution, Institute for Advanced Studies in Multidisciplinary Science and Technology, East China Normal University, Shanghai, China; 2 School of Biological and Chemical Sciences, Queen Mary University of London, London, United Kingdom; University of Oxford, United Kingdom

## Abstract

Closely related taxa living in sympatry provide good opportunities to investigate the origin of barriers to gene flow as well as the extent of reproductive isolation. The only two recognized subspecies of the Chinese rufous horseshoe bat *Rhinolophus sinicus* are characterized by unusual relative distributions in which *R. s. septentrionalis* is restricted to a small area within the much wider range of its sister taxon *R. s. sinicus*. To determine the history of lineage divergence and gene flow between these taxa, we applied phylogenetic, demographic and coalescent analyses to multi-locus datasets. MtDNA gene genealogies and microsatellite-based clustering together revealed three divergent lineages of *sinicus*, corresponding to Central China, East China and the offshore Hainan Island. However, the central lineage of *sinicus* showed a closer relationship with *septentrionalis* than with other lineages of *R. s. sinicus*, in contrary to morphological data. Paraphyly of *sinicus* could result from either past asymmetric mtDNA introgression between these two taxa, or could suggest *septentrionalis* evolved *in situ* from its more widespread sister subspecies. To test between these hypotheses, we applied coalescent-based phylogenetic reconstruction and Approximate Bayesian Computation (ABC). We found that *septentrionalis* is likely to be the ancestral taxon and therefore a recent origin of this subspecies can be ruled out. On the other hand, we found a clear signature of asymmetric mtDNA gene flow from *septentrionalis* into central populations of *sinicus* yet no nuclear gene flow, thus strongly pointing to historical mtDNA introgression. We suggest that the observed deeply divergent lineages within *R. sinicus* probably evolved in isolation in separate Pleistocene refugia, although their close phylogeographic correspondence with distinct eco-environmental zones suggests that divergent selection might also have promoted broad patterns of population genetic structure.

## Introduction

Closely related taxa that have sympatric or parapatric ranges provide good opportunities to investigate the origins of barriers to gene flow and the extent of reproductive isolation. Although gene flow has a powerful homogenizing effect that can hamper the divergence of populations into new taxa [1] by preventing the evolution of strong reproductive isolation [2], several recent studies suggest divergence with gene flow is not uncommon, not only in the process of speciation but also after speciation is complete [3], [4], [5], [6]. Indeed there is good evidence that introgressive hybridization may occur between closely related species on secondary contact after a period of allopatric isolation [7], [8]. By allowing for genetic exchange during divergence, the isolation-with-migration (IM) model has been developed to disentangle the relative importance of complete isolation and divergence with gene flow in the process of taxon divergence [9].

As with many temperate taxa, tropical and subtropical species underwent radical range shifts during Pleistocene glacial-interglacial cycles [10], [11], [12]. Recent studies have shown that Pleistocene climatic fluctuations and associated sea level changes have contributed to intraspecific diversification of many taxa from East Asia [12], [13]. Several refugia have been reported in the east and southwestern plateau of China [13], [14], [15], which appear to have promoted population divergence and speciation [10]. In addition to global climate cycles, the biota of East Asian was further impacted by severe climatic and environmental changes due to the uplift of the Qinghai-Tibetan Plateau (QTP). This process not only dramatically modified the topography of East Asia, leading to the current higher elevations in the west [16], but also gave rise to the East-Asia monsoon, which further enhanced the climatic difference between the east and south-west [16].

**Figure 1:**
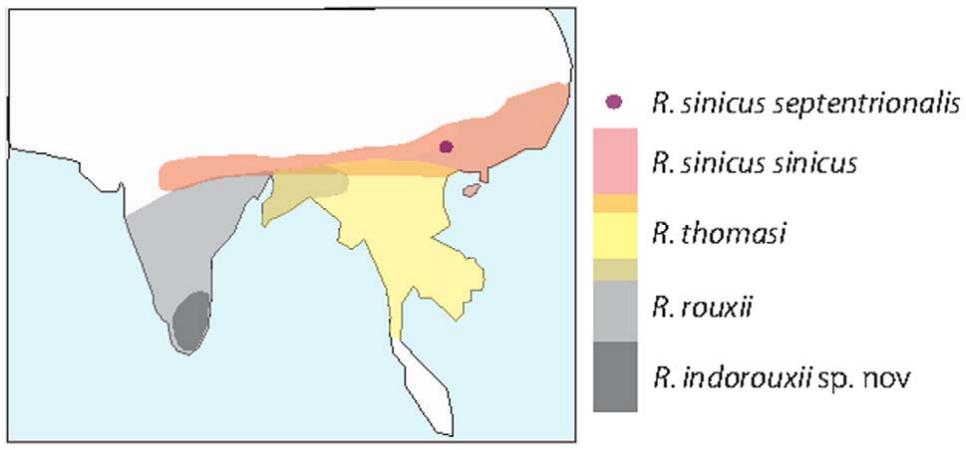
Map showing the distributions of the member species of the *R. rouxii*-group, and the ranges of the focal subspecies of *Rhinolophus sinicus*.

The horseshoe bats (family Rhinolophidae) number over 70 species and show highest diversity in the Old World tropics and subtropics [17]. The Chinese rufous horseshoe bat *Rhinolophus sinicus* is a common species in East Asia with a wide longitudinal range that encompasses the Qinghai-Tibetan Plateau. Its sister species, *R. thomasi* and *R. rouxii,* occur further south and west, respectively, and together with a recently described cryptic species of *R. rouxii* from South India (*R. indorouxii* sp. nov) [18], these taxa constitute the only members of the so-called *rouxii*-group [17] (also see Figure 1). Previously, *R. sinicus* was regarded as a subspecies of *R. rouxii* based on craniodental features [19], but was later elevated to a distinct species following detailed phenetic, molecular [20] and cytogenetic analyses [21], [22].

**Figure 2:**
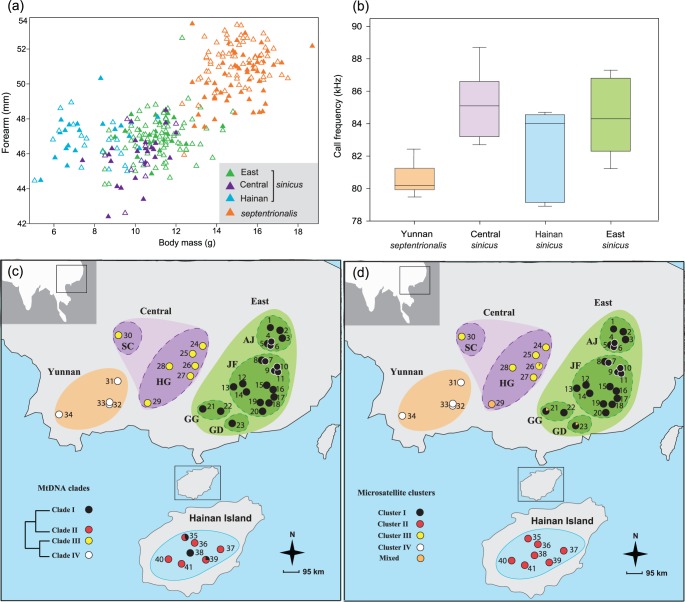
Morphological and echolocation data for bats with corresponding membership in mtDNA clades and microsatellite clusters. (a) Forearm (mm) and body mass (g) data measured for 396 individuals. Open and filled triangles correspond to female and male bats, respectively. (b) Boxplots of the echolocation call frequency (kHz) recorded from 130 individuals. (c) Map showing the sample sites of *R. sinicus* in this study. Forty-one sampling localities were classified into eight regions, depicted by dashed lines. These regions were further divided into four groups coded by colours (East: green; Central: purple; Hainan Island (HND): blue; Yunnan (YN): orange). Populations are presented as pie charts in which individuals are coloured based on the membership of mtDNA clades (I, II, III and IV). The relationships between clades are shown on the left side of the map (see details in Figure 2). (d) Populations are presented as pie charts in which individuals are coloured based on the membership of microsatellite clusters (I, II, III, IV and mixed, see Figure 4).

In spite of its wide distribution, *R. sinicus* is currently recognized as consisting of just two subspecies (see Figure 1): *R. s. sinicus* and *R. s. septentrionalis*, which can be distinguished on the basis of morphological characters; in particular, *septentrionalis* has a larger body size than *sinicus* (see [17] and Figure 2a in this study). These two recognized subspecies have unusual relative distributions that raise questions about the origin of *septentrionalis*. Currently the subspecies *sinicus* is thought to range from the Himalayas, through northern Vietnam and regions of Sichuan to southeastern China. In contrast, *septentrionalis* is thought to be highly restricted to Yunnan Province in China [17] and is completely surrounded by its more widespread sister taxon (see Figure 1). Three alternative hypotheses might account for the unusual asymmetric distributions of these two taxa. The first and most simple explanation is that *septentrionalis* and *sinicus* are not true sister taxa and thus the current taxonomy is incorrect. Second, they are sister taxa and *septentrionalis* has evolved *in situ* via divergence from *sinicus*, in which case the former will appear to be derived from the latter in phylogenetic analyses, and will harbor a subset of the genetic variation. If this is true we might also expect historical gene flow during divergence. The third hypothesis is that *septentrionalis* evolved allopatrically, possibly related to Pleistocene glaciation and/or uplift of the Qinghai-Tibetan Plateau. In this scenario, the current sympatric distributions will have arisen from secondary contact.

To distinguish between these three broad hypotheses, we sampled individuals of *R. sinicus* from across China, including both putative subspecies, and generated multilocus datasets consisting of two mitochondrial genes, five nuclear genes and nine microsatellite loci. We then conducted a range of phylogenetic, demographic and coalescent analyses that together provide information on the history of lineage divergence and gene flow among these taxa.

## Materials and Methods

### Ethics Statement

Our procedure of sampling consisted of taking wing membrane biopsies from animals, and was approved by the National Animal Research Authority, East China Normal University (approval ID 20080209). Bats were captured using mist nets at cave exits, and the bats were measured, sampled and subsequently released *in situ*.

### Bat Capture and Measurement, Sample Collection and DNA Extraction

We collected data from individuals of *R. sinicus* from 41 localities across the Chinese mainland, including the known range of *septentrionalis* in Yunnan Province, and the offshore Hainan Island (Figure 2c and Table 1). Bats were captured in mist nets set at cave exits and, for each individual, a wing membrane biopsy was taken and stored in 95% ethanol at −20°C until genomic DNA was extracted using Qiagen kits. Additionally, the forearm of each individual was measured with dial calipers, and the body mass obtained using Pesola spring scales. For a subset of bats, we also recorded the echolocation call resting frequency while in the hand using the Avisoft UltraSoundGate 116Hnb kit (Avisoft, Berlin, Germany) connected to a laptop. Spectrograms were analysed using Avisoft-SASLab Pro software (Avisoft) and the constant frequency of the second harmonic was extracted from a series of three consecutive pulses.

**Table 1:** Summary of the number and sampling locality of individuals used in the molecular analyses.

No	Locality	Coordinates	Code	*Cytb*	*ND1*	*Chd1*	*SWS1*	*THY*	*USP9x*	Microsatellites
1	Qingyang, Anhui	N30∶20:511 E117∶50:128	AJ	1	1	1	1	1	1	1
2	Jingxian, Anhui	N30∶26:785 E118∶24:783	AJ	6	2	1	2	1	1	6
3	Huashanmiku, Anhui	N29∶45:199 E118∶23:331	AJ	7	2	4	2	1	1	11
4	Sanling mountain, Jiangxi	N29∶22:112 E117∶34:324	AJ	8	1	6	2	1	1	12
5	Qingfeng cave, Jiangxi	N29∶22:262 E117∶39:357	AJ	5	1	1	1	1	1	5
6	Qinhui cave, Jiangxi	N29∶22:662 E117∶32:335	AJ	5	1	2	–	1	1	6
7	Longhu mountain, Jiangxi	N28∶04:107 E116∶58:227	JF	5	1	2	2	1	1	6
8	Lijia country, Jiangxi	N28∶06:726 E116∶59:282	JF	8	1	5	3	1	1	13
9	Wuyishan baohuqu, Fujian	N27∶44:000 E117∶40:000	JF	10	2	9	5	2	2	26
10	Wuyishan tiliqiao, Fujian	N27∶44:543 E117∶29:953	JF	1	–	1	1	1	1	2
11	Wuyishan Yanzijiao, Fujian	N27∶48:511 E117∶42:505	JF	1	1	1	1	–	–	2
12	Taihe, Jiangxi province	N26∶36:151 E114∶12:734	JF	8	2	3	2	–	1	16
13	Jinggang mountain, Jiangxi	N26∶31:215 E115∶06:610	JF	2	1	2	–	1	1	2
14	Xingguo, Jiangxi	N26∶19:314 E115∶35:229	JF	3	2	3	1	1	1	5
15	Taining, Fujian	N26∶42:236 E117∶29:867	JF	4	1	5	4	1	1	11
16	Jiangle, Fujian	N26∶39:537 E117∶34:387	JF	4	2	2	1	1	2	4
17	Mingxi, Fujian	N26∶21:440 E117∶11:398	JF	2	1	–	–	1	1	2
18	Yongan, Fujian	N25∶51:500 E117∶17:000	JF	1	1	1	–	1	1	2
19	Liancheng, Fujian	N25∶12:404 E117∶15:066	JF	4	2	3	2	2	2	4
20	Shanghang, Fujian	N25∶15:020 E116∶49:009	JF	2	1	2	1	1	1	4
21	Guilin, Guangxi	N25∶16:278 E110∶17:009	GG	8	2	2	3	4	6	10
22	Ruyuan, Guangdong	N24∶59:086 E113∶08:523	GG	1	1	–	1	1	1	1
23	Luofushan, Guangdong	N23∶15:589 E114∶03:656	GD	5	2	6	3	3	2	6
24	Zhangjiajie, Hunan	N29∶21:410 E110∶34:783	HG	7	2	1	2	1	2	10
25	Yongshun, Hunan	N29∶03:720 E109∶38:358	HG	1	1	–	–	2	2	4
26	Jishou, Hunan	N28∶18:208 E109∶39:175	HG	10	3	2	1	3	3	12
27	Fenghuang, Hunan	N27∶59:580 E109∶33:786	HG	1	1	–	–	–	1	2
28	Wuchuan, Guizhou	N28∶34:237 E107∶54:058	HG	2	1	1	1	1	–	1
29	Anlong, Guizhou	N25∶16:577 E105∶31:931	HG	2	1	1	1	1	1	1
30	Emeishan, Sichuan	N29∶34:803 E103∶24:708	SC	3	2	3	2	2	2	3
31	Huize, Yunnan	N26∶42:000 E103∶30:000	YN	7	1	1	1	2	1	11
32	Jiuxiang, Yunnan	N25∶07:000 E103∶22:000	YN	1	1	1	–	1	1	1
33	Fumin, Yunnan	N25∶11:796 E102∶27:863	YN	3	–	–	–	2	–	3
34	Yongde, Yunnan	N24∶21:427 E099∶02:161	YN	10	6	5	3	9	4	11
35	Yinggeling, Hainan	N19∶04:982 E109∶33:107	HND	7	2	8	9	3	2	19
36	Wuzhishan, Hainan	N18∶46:309 E109∶31:012	HND	2	1	3	3	1	1	3
37	Qiongzhong, Hainan	N18∶49:589 E110∶00:435	HND	–	–	3	3	2	2	13
38	Baoqing, Hainan	N18∶42:237 E109∶41:528	HND	1	1	1	1	1	1	3
39	Lingshui, Hainan	N18∶38:586 E109∶57:636	HND	3	3	1	1	1	1	5
40	Jianfengling, Hainan	N18∶47:275 E108∶57:364	HND	1	1	1	–	1	1	1
41	Maogan, Hainan	N18∶36:306 E109∶26:776	HND	3	1	–	–	–	–	3
	Total			165	59	94	66	61	56	263

The taxonomic status of each individual was initially established on the basis of forearm length following reference [17], and validated retrospectively with molecular data and echolocation call frequencies (see Results). This led to us classifying the bats into four geographical groups, each containing several localities as follows: East (AJ, JF, GG and GD), Central (SC and HG), Hainan Island (HND) and Yunnan (YN), of which the first three were *sinicus* and the latter was *septentrionalis* (also Figure 2c).

### DNA Sequencing and Phylogenetic Analyses

We amplified and sequenced two mitochondrial genes (*Cytb* and *ND1*), three autosomal (*Chd1*, *SWS1* and *THY*), one X-chromosomal (*USP9x*) and one Y-chromosomal (*Dbx*) genes. Information on the samples and primers for these markers are summarized in Table 1 and Table 2, respectively. PCR was performed in 50 µl reaction mixtures containing 10–50 ng DNA, 0.25 mM of each primer and 25µl Premix Taq polymerase (TaKaRa). The thermal profile for each marker has been described previously (see references in Table 2). PCRS were carried out on a PTC-220 thermal cycler (Bio-Rad).

**Table 2:** Details of primers used in this study.

Molecular markers	ID	Length(bp)	Primers (5′->3′)	Reference
Cytochrome *b*	*Cytb*	491	F: TAGAATATCAGCTTTGGGTG	[68]
			R: AAATCACCGTTGTACTTCAAC	
NADH dehydrogenase 1	*ND1*	957	F: CCTCGATGTTGGATCAGG	[69]
			R: GTATGGGCCCGATAGCTT	
Nucleosome remodeling factor	*Chd1*	424	F:GATAARTCAGARACAGACCTTAGACG	[70]
			R: TTTGGCATTCACCTGYACTCC	
Short-wavelength-sensitive opsin	*SWS1*	510	F: CACAGGCTATGGTGCTGACTT	[62]
			R: GCCCGTGGGGATGGCTATTGA	
Thyrotropin	*THY*	381	F: GGGTATGTAGTTCATCTTACTTC	[71]
			R: GGCATCCTGGTATTTCTACAGTCTTG	
Ubiquitin specific protease 9 X	*USP9x*	591	F: GGCAGACAGGTTGATGAC TTGGA	[70]
			R: AGGTCTGCAACTTGC CAAAGG AA	
DEAD box RNA helicase Y	*Dby*	234	F: GGTCCAGGAGARGCTTTGAA	[70]
			R: CAGCCAATTCTCTTGTTGGG	

For sequencing, we used the forward primer of each mitochondrial gene and both primers of each nuclear gene. The products were analysed on an ABI PRISM 3700 automated sequencer (Applied Biosystems). Heterozygous sites in the nuclear gene sequences were identified by the presence of clear double overlapping peaks in both the forward and reverse sequence chromatograms. Haplotypes were resolved unambiguously if there was only one heterozygous site, and, for sequences with more than one heterozygous site, we resolved haplotypes probabilistically using PHASE version 2.1 [23]. The sequences were aligned using CLUSTAL_X 1.83 [24] and edited by eye with BIOEDIT version 7.0.0 [25]. All sequences obtained in this study were deposited in GenBank (accession numbers: JN650618–JN651091 and JN651094–JN651146). For each region the number of haplotypes, haplotype diversity and nucleotide diversity was calculated using DnaSP 4.0 [26].

Phylogenetic analyses were performed for both *Cytb* and concatenated mtDNA sequences (*Cytb* and *ND1*). To investigate the relationship between the two recognized subspecies of *R. sinicus* we also incorporated *Cytb* data from two outgroups: *R. thomasi* from the *rouxii*-group, and *R. affinis* from a different clade (accession numbers FJ85215 and DQ297582 respectively). To establish phylogenetic consensus we implemented maximum parsimony (MP) and neighbor joining (NJ) in PAUP* 4.0b10 [27], Bayesian Inference (BI) in MrBayes 3.1.2 [28] and maximum-likelihood (ML) in PhyML [29]. For MP, we used heuristic searching with tree-bisection-reconnection and branch swapping. Characters were treated as equally weighted and unordered. Node support was estimated based on 1000 bootstraps. We used MODELTEST 3.0 [30] to determine the best-fit substitution models to be HKY+G [G = 0.092] for *Cytb* and HKY+G [G = 0.102] for concatenated mtDNA. For BI, we performed two simultaneous runs of Metropolis-coupled Markov chain Monte Carlo (MCMC) analysis using the chosen molecular evolution model parameters, each comprising four chains and 10 million generations. Trees and parameters were sampled every 100 generations, and the first 25% of the sampled trees were discarded as a burn-in. For NJ and ML, node supports were estimated based on 1000 bootstraps. The best topology was compared with several alternative candidate hypotheses using PAUP and CONSEL [31]. Log-likelihoods of site-pattern trees estimated with PAUP were used by CONSEL to calculate the P-values for several statistical tests for which only the Approximately Unbiased (AU) test and Shimodaira-Hasagawa (SH) test [32] are presented here.

At the intraspecific level, bifurcating trees may not be the most appropriate way to represent genealogical relationships among haplotypes [33], and therefore, we also constructed networks based on *Cytb* and concatenated mtDNA haplotypes using the maximum parsimony method implemented in TCS version 1.21 [34]. Ambiguous connections were resolved based on the criteria of topology, frequency and geography [35].

To estimate the time to the most recent common ancestor (TMRCA) for each mtDNA (*Cytb*) clade (see Figure S2), we used a Bayesian MCMC approach implemented in BEAST version 1.4.6 [36]. A HKY+G [G = 0.01] substitution model for *Cytb* dataset without outgroup estimated by MODELTEST, and a relaxed-clock model with an uncorrelated lognormal distribution for the substitution rate, were applied. We used a substitution rate of 0.02/Myr based on a divergence rate of 4%/Myr for *Cytb* obtained for *Pipistrellus* bats [37]. We performed two independent runs of 10^7^ generations, each with a burn-in of 10^6^ generations, and sampled every 1000 steps. These two runs were then combined in TRACER version 1.4 [38], which were also used to examine the effective sample size (ESS) for each parameter.

For each nuclear gene, phylogenetic relationships among reconstructed haplotypes were displayed using haplotype networks. To infer the species phylogeny from these multiple nuclear sequences, as well as infer each gene tree, we used the Bayesian approach in BEST 2.0 that takes account the coalescent process [39], [40]. For this analysis, run at the Computational Biology Service Unit at Cornell University, model parameters for each marker were estimated using MODELTEST. The priors for theta were an inverse gamma distribution (3, 0.003) and the priors for gene mutation were a uniform distribution (0.5, 1.5). We performed two runs of Metropolis-coupled MCMC, each comprising four chains and 20 million generations. Trees and parameters were sampled every 100 generations, and the first 25% of the sampled trees were discarded as a burn-in. A consensus tree was constructed from the estimated posterior distribution and used as the estimated species tree [39].

### Hypothesis Testing of Lineage Divergence

To test probabilistically among alternative hypotheses for the history of lineage divergence, we used the approximate Bayesian computation procedure (ABC, [41]) in DIY ABC v1.0.4.37 [42]. ABC analyses were applied to sequences of the concatenated mtDNA data as well as three informative nuclear genes (*THY* was excluded because it contains indels and has less informative sites). We tested the following three competing scenarios: (1) *septentrionalis* arose recently via divergence from Central *sinicus* following a more ancient divergence between the common ancestor of these taxa, and lineages of *sinicus* from the East and Hainan. We also incorporated a bottleneck early in the history of *septentrionalis*, because of its small population size. This scenario corresponds approximately to our hypothesis 2 in the Introduction. Scenario (2) is the same as (1) but without the bottleneck. In scenario (3) *septentrionalis* was considered the ancestor of all *R. sinicus* with a subsequent divergence between Central *sinicus* and East/Hainan *sinicus* (see details in Figure S1). In the first scenario, a population size with the prior of 10–10^4^ individuals and with a bottleneck of 1 to 1000 generations was used as the founding population for *septentrionalis*. A total of 3 million datasets were simulated, of which the 1% closest to the observed data were used to estimate the relative posterior probabilities of each scenario via a logistic regression approach [43]. Equal prior probability was set for each scenario.

### Microsatellite Genotyping

Nine primer pairs developed for *R. sinicus* [44] were used to screen a total of 263 bats. PCRs were undertaken in 15 µl reaction volumes (containing 50–100 ng genomic DNA, 0.25 mM forward primer (labeled with FAM, HEX or TAMRA), 0.25 mM unlabeled reverse primer, 0.2 mM of each dNTP, 0.2 U Hotstar Taq DNA polymerase (Qiagen) and 1.5 mM MgCl_2_) with the thermal profile: initial denaturation step at 95°C for 15 min, followed by 35 cycles of 30 s at 94°C, 30 s at (55–66°C) and 30 s at 72°C and final extension step at 72°C for 20 min. Sample information for each locality is summarized in Table 1. The products were visualized on an ABI 3730 sequencer and analyzed with GeneMapper v3.7 (ABI).

Only individuals genotyped at over seven loci were used in subsequent analyses. Data were checked for possible null alleles and genotyping errors with Micro-checker v2.2.3 [45]. GENEPOP v3.4 [46] was used to test for deviation from Hardy–Weinberg equilibrium (HWE) and linkage equilibrium (LE) among all loci across all populations. To quantify genetic diversity per region, we calculated allelic richness (standardized for n = 3) using FSTAT [47], and we calculated the mean number of alleles per locus, the number of private alleles and expected and observed heterozygosity using GDA [48].

### Microsatellite-based Population Genetic Analyses

To assess the relationship among populations, we used STRUCTURE 2.2 [49] to assign individuals to clusters, for cases of 2 to 8 clusters (K) in the data, the upper limit corresponding to the number of regions in the study data [12], [14]. We performed ten replicate runs (10^6^ iterations and with an initial burn-in of 10^5^ iterations per run) and used the admixture model. The most likely number of clusters was inferred based on the method described in [50]. Similar replicate runs were grouped based on the symmetric similarity coefficient of >0.9 using the Greedy algorithm in CLUMPP [51] and visualized using DISTRUCT 1.1 [52]. To complement the results of STRUCTURE, we used GENELAND [53] and TESS [54], which can incorporate the spatial coordinates of individuals when uncovering population structure. For both analysis, we performed ten independent runs from K = 2 to 8, with 10^6^ iterations and a burn-in of 10%.

Finally, we repeated ABC analyses for microsatellite data, again using DIY ABC v1.0.4.37 [42]. Details of the scenarios tested are described above and shown in Figure S1. In this analysis, a total of 1.5 million datasets were simulated, of which the 1% closest to the observed data were used to estimate the relative posterior probabilities of each scenario via a logistic regression approach.

### Estimates of Gene Flow

To test for gene flow between *septentrionalis* and adjacent populations of *sinicus*, we used the isolation with migration (IM) model [9] implemented in the program IM [55]. The IM model assumes that the populations under study are panmictic, and that the markers are selectively neutral and free from recombination. We tested for recombination by calculating the minimum number of recombination events (Rm) based on four-gamete test [56] and we tested for neutrality using the McDonald–Kreitman test [57] and the Hudson–Kreitman–Aguade test [58]. These analyses were conducted in DnaSP. We did not detect deviation from neutral expectations for all markers; however, there was some evidence of recombination for sections of sequence of *Cytb*, *Chd1* and *SWS1*, and only nonrecombined regions were used in the subsequent IM analyses.

IM analyses were performed for four datasets: (i) mtDNA (*Cytb*), (ii) combined ncDNA (*USP9x*, *Chd1*, *SWS1* and *THY*), (iii) combined mtDNA and ncDNA, and (iv) microsatellites. To incorporate differences in the effective population size of the markers, we set inheritance scalars as follows: 0.25 for *Cytb*, 0.75 for the X-linked locus *USP9x* and 1 for the three other nuclear genes (*Chd1*, *SWS1* and *THY*) and also for the microsatellites. Scaled directional migration rates (*m_1_* =  m_1_/u and *m_2_* =  m_2_/u; u is mutation rate per locus per year) were estimated between *septentrionalis* and adjacent populations of *sinicus*. Preliminary runs with large parameter intervals were used to determine the starting values of prior distributions for the subsequent runs. Three final runs were conducted with different random seeds and a single chain with a burn-in of 10^7^ steps for 2×10^8^ iterations. For the sequence datasets, each run included five MCMCs with ten multiple chain-swapping attempts, and a geometric heating scheme with the heating terms h1 set to 0.05. For the microsatellites dataset, each run included eight MCMCs with 25 multiple chain-swapping attempts, and a geometric heating scheme with the heating terms h1 and h2 set to 0.05 and 2.

## Results

In total, we captured and measured 396 bats, of which we recorded the echolocation calls of 130 individuals and undertook genetic analyses on 263 individuals.

### Morphological Data and Echolocation Call Frequency

Individual bats initially assigned to *sinicus* showed similar body size and echolocation call frequencies across the study region, with considerable overlap among bats captured from the regions Central, East and Hainan Island. In contrast, bats captured in Yunnan Province around the type locality of *septentrionalis* were characterized as having larger forearms and lower echolocation call frequencies than bats from neighbouring areas, consistent with the presence of a distinct taxon in this area. For a summary of these findings see Figure 2a and b.

### Mitochondrial DNA Sequences

The alignment of *Cytb* (491 bp) from 165 individuals contained 56 haplotypes with 66 polymorphic sites, and the alignment of *ND1* (957 bp) from 59 individuals contained 28 haplotypes with 88 polymorphic sites. No premature stop codons were observed in either of these genes, suggesting they are functional mitochondrial genes rather than nuclear copies. When concatenated, the mtDNA alignment spanned 1448 bp, and data from 59 individuals contained 40 haplotypes with 124 polymorphic sites.

**Figure 3:**
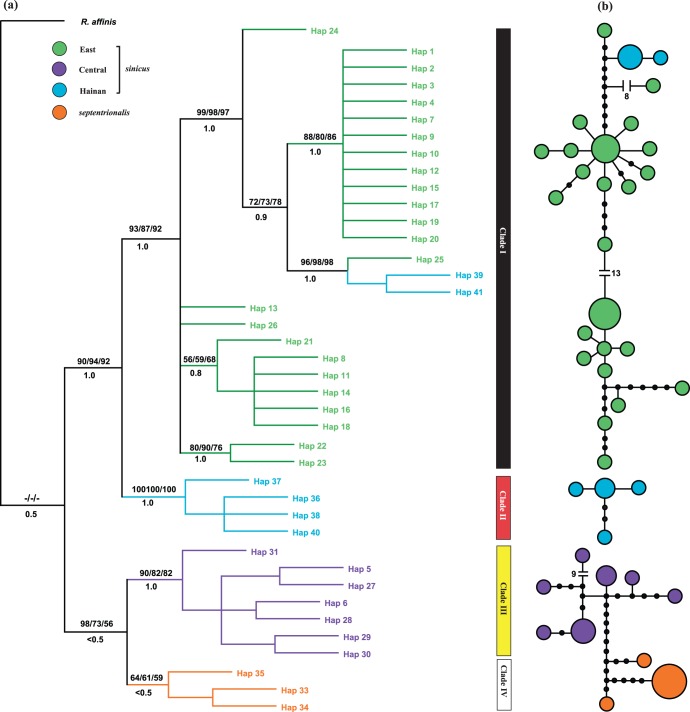
Maximum parsimony tree and statistical parsimony network based on concatenated mtDNA sequences. (a) The phylogenetic tree. Node support is indicated with MP, NJ and ML bootstrap values and Bayesian posterior probabilities (given above and below the branch, respectively). (b) The network with a 95% connection limit. Each circle represents a single haplotype and the circle size is scaled by the haplotype frequency. Filled black circles represent missing or unsampled haplotypes. The numbers in the network represent mutational steps between haplotypes.

Phylogenetic tree and network analyses indicated that *R. sinicus* is a monophyletic grouping to the exclusion of the closely related congener *R. thomasi* (see Figure S2), supporting the sister relationship of the two subspecies *sinicus* and *septentrionalis*. Within *R. sinicus*, all tree-building methods recovered similar topologies in which four well-supported clades were resolved, corresponding closely to East, Hainan Island, Central and Yunnan, hereafter referred to as clades I, II, III and IV, respectively (Figures 2 and 3). Two haplotypes from Hainan (clade II) were exceptions to this trend, and were classified with haplotypes from the East (clade I). Surprisingly, *sinicus* bats from the Central area showed a closer relationship with *septentrionalis* than with other *sinicus* lineages. The AU and SH tests both supported the current topology and were able to reject the alternative phylogenetic hypothesis ([(IV,(III,II,I))]; AU test, P<0.001; SH test, P = 0.03). Consistent with the tree results, network analysis for concatenated mtDNA recovered three networks with a 95% connection limit, corresponding to clade I, II and III+IV, respectively (Figure 3).

For the *Cytb* dataset, the samples from Yunnan contained the lowest level of nucleotide diversity followed by those from the Central area (Table 3). In contrast, the samples from Hainan and the East showed very high levels of nucleotide diversity, indicating a long and stable evolutionary history or just a mixture of different evolutionary lineages. The latter scenario is supported by the observation of divergent lineages within the East samples and of several haplotypes seen in Hainan samples also nested within the East clade.

**Table 3:** Summary of genetic diversity for five gene sequences and eight microsatellite loci in eight regions of *R. sinicus*.

Genes	Parameters\regions	AJ	JF	GG	GD	East	HG	SC	Central	HND	YN
*Cytb*	N1	32	55	9	5	101	23	3	26	17	21
	n	8	19	4	4	32	8	3	11	7	5
	h	0.724	0.893	0.694	0.900	0.902	0.581	1.000	0.674	0.772	0.681
	π(%)	0.214	0.674	0.328	0.855	0.652	0.328	0.407	0.449	1.480	0.376
*Chd1*	N2	15	38	2	6	61	4	3	7	17	7
	n	2	2	1	2	2	1	1	1	2	2
	h	0.495	0.483	–	0.571	0.480	–	–	–	0.382	0.286
	π(%)	0.117	0.114	–	0.135	0.113	–	–	–	0.992	0.067
*SWS1*	N3	9	23	4	3	39	5	2	7	17	4
	n	8	13	3	2	18	5	2	7	7	4
	h	0.956	0.913	0.833	0.667	0.911	1.000	1.000	1.000	0.772	1.000
	π(%)	0.643	0.621	0.295	0.131	0.584	2.630	0.984	2.110	0.402	2.940
*THY*	N4	6	14	5	3	28	8	2	10	8	14
	n	2	2	2	1	3	5	2	6	2	5
	h	0.286	0.366	0.524	–	0.340	0.833	0.667	0.803	0.530	0.450
	π(%)	0.075	0.096	0.150	–	0.092	0.656	0.175	0.537	0.140	0.128
*USP9x*	N5	6	16	7	2	31	9	2	11	8	6
	n	1	2	6	1	6	6	2	6	1	1
	h	–	0.125	0.952	–	0.488	0.889	1.000	0.873	–	–
	π(%)	–	0.021	0.354	–	0.123	0.291	0.338	0.283	–	–
	Mean π(%)	–	–	–	–	0.228	–	–	0.733	0.384	0.784
Microsatellites	N6	40	95	11	6	152	30	3	33	43	23
	A	6.4	9.5	5.6	4.8	11.9	9.8	3.5	12.3	8.9	10.0
	H_E_	0.655	0.645	0.677	0.627	0.673	0.759	0.700	0.759	0.698	0.807
	H_O_	0.653	0.625	0.693	0.583	0.636	0.713	0.625	0.705	0.680	0.750
	R_S_	3.15	3.18	3.36	3.24	3.36	3.99	3.63	3.98	3.47	4.06
	Private alleles	3(0)	5(0)	2(1)	0	–	12(1)	3(3)	–	5(0)	8(3)

N1–N6, sample sizes for genes and microsatellite loci; n, number of haplotypes observed; h, haplotype diversity; π(%), nucleotide diversity; mean π(%), mean nucleotide diversity of four nuclear genes. A, mean number of alleles per locus; H_E_, expected heterozygosity; H_O_, observed heterozygosity; Rs, allele richness standardized by three individuals. The numbers of private alleles with >10% frequency are shown in parentheses.

Bayesian estimates of the TMRCA performed in BEAST provided reliable estimates of all parameters with ESS >500. The inferred TMRCA for all *Cytb* sequences was around 1.5 MYA, corresponding to the early Quaternary. TMRCA estimates obtained for all *septentrionalis* (clade VI) and for all Central *sinicus* (clade III) were similar to each other, both estimated to be around 0.3 MYA. This date was more recent than that of the East *sinicus* (clade I) but older than Hainan (clade II) (see details in Table 4).

**Table 4:** Mean estimates of TMRCA with 95% credible intervals for each clade or group of clades based on *Cytb* data.

Clade	Mean (Myr)	95% C.I. (Myr)
clade I	0.48	0.24–0.78
clade II	0.18	0.05–0.37
clade I +II	0.99	0.53–1.57
clade III	0.33	0.16–0.52
clade IV	0.32	0.12–0.53
clade III+IV	0.48	0.23–0.76
clade I+III+IV	1.30	0.72–2.07
clade II+III+IV	1.20	0.64–1.94
clade I+II+III+IV	1.51	0.84–2.42

### Nuclear DNA Sequence Analyses

Within *R. sinicus* all nuclear markers were polymorphic except for *Dby* which was invariant across the bats screened here and, therefore, discarded prior to genetic analyses. The alignment of *Chd1* (424 bp) from 94 individuals included 5 haplotypes based on 4 polymorphic sites and 11 indels. The alignment of *SWS1* sequences (510 bp) from 66 individuals included 33 haplotypes based on 32 polymorphic sites and 20 indels. The alignment of *THY* (381 bp) from 61 individuals included 12 haplotypes based on 6 polymorphic sites and 10 indels. The alignment of *USP9x* sequences (591 bp) from 56 individuals included 12 haplotypes based on 12 polymorphic sites. Levels of nucleotide diversity were not consistent across four ncDNA markers (see Table 3). Overall, Yunnan and the Central area showed the highest mean levels of nucleotide diversity (π(%), 0.784 and 0.733, respectively). The levels recorded in the East were significantly lower (0.228; t = −4.667, df = 3, P = 0.018), and the Hainan population was also lower than the population from Yunnan, although this was not significant (0.384; t = −1.826, df = 3, P = 0.165).

**Figure 4:**
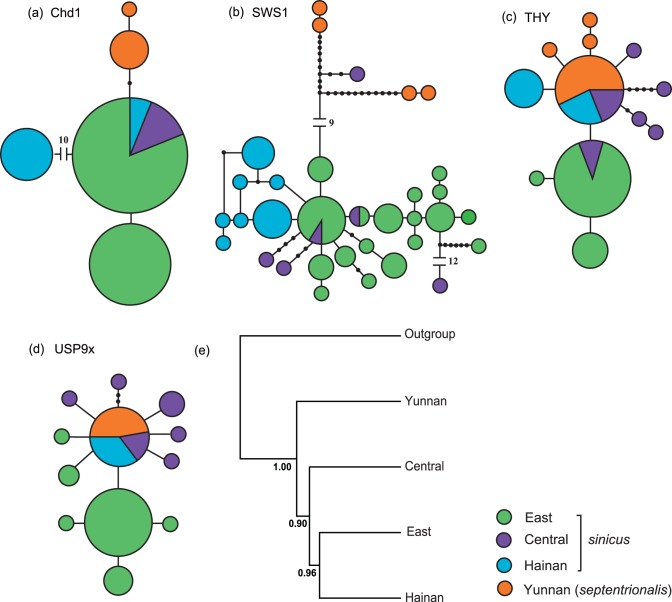
Statistical parsimony networks for four nuclear markers and species tree. Networks for *Chd1* (a), *SWS1* (b), *THY* (c) and *USP9x* (d). Each circle in the network represents a single haplotype and the circle size is scaled by the haplotype frequency. Filled black circles represent missing or unsampled haplotypes. The numbers in the network represent mutational steps between haplotypes. (e) Species tree of *R. sinicus* lineages and outgroups estimated on the basis of four nuclear genes. Node support is indicated with posterior probabilities.

Haplotype networks reconstructed for *Chd1* and *SWS1* both resolved two sub-networks, broadly corresponding to *sinicus* and *septentrionalis* (Figure 4a and b). The exception is that one *SWS1* haplotype from Central *sinicus* showed a closer relationship to *septentrionalis* haplotypes than to other haplotypes of *sinicus*. Between the sub-networks there were two transversions in the *Chd1* network and over ten mutational steps in the *SWS1* network. Within *sinicus*, haplotypes of Hainan were seen to be very divergent from those from the East and Central; specifically, in the *Chd1* network, most individuals from Hainan were separated from those of other *sinicus* with ten indels, while in the *SWS1* network all Hainan haplotypes clustered together. In contrast to *Chd1* and *SWS1*, the network analysis for *THY* and *USP9x* did not recover clear subspecies, and instead, individuals of *septentrionalis* were mixed with Central and Hainan *sinicus* (Figure 4c and d).

The species tree estimated from the four nuclear markers in BEST supported the reciprocal monophyly of *septentrionalis* and *sinicus* with high node support (Figure 4e). Moreover, East *sinicus* showed a closer relationship with Hainan *sinicus* than with Central *sinicus,* consistent with the mtDNA tree.

### Microsatellite-based Population Genetic Analyses

No evidence for allele dropout and scoring error due to stuttering was detected using Micro-checker except for one locus (SM24) which was deleted in the subsequent analysis. We detected no significant departure from Hardy-Weinberg equilibrium after Bonferroni correction, although significant departure from linkage equilibrium was detected between SF77 and SN30 in several locations of AJ and JF.

Genetic diversity decreased broadly from the west (i.e. Yunnan and the Central area) to the east (i.e. East and Hainan) (see Table 3). Private alleles with the frequency over 10% were observed in GG, HG, SC and YN. Among them, one allele with a frequency of 28% in Yunnan was private to *septentrionalis*, and thus private to this subspecies.

**Figure 5:**
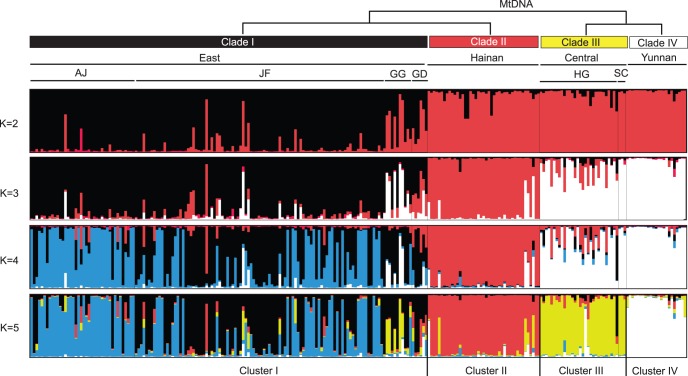
Clustering of *R. sinicus* individuals in STRUCTURE based on microsatellite genotypes. Clusters are shown for values of K = 2 to 5 inclusive. The mtDNA clade relationships are shown above the four cluster plots.

Clustering at a range of values of K using STRUCTURE not only revealed substantial phylogeographic structure but also recovered hierarchical relationships among populations. Individuals of East *sinicus* were firstly separated from others at K = 2 (Figure 5), so conflicted with mtDNA and ncDNA sequence analyses in which Hainan showed a closer relationship with East *sinicus* than with Central *sinicus*+*septentrionalis* (Figures 3 and 4). This subdivision showed the highest value of ΔK (Figure S3). However, further substructuring was observed when increasing the value of K. Specifically, at K = 3, Hainan was separated from Central *sinicus*+*septentrionalis*. Further clustering was observed within East at K = 4. At K = 5, *septentrionalis* and Central *sinicus* finally became separate clusters. When increasing the value of K, further clusters were only detected in East *sinicus* but the clustering patterns were generally uninformative. Clustering conducted in GENELAND and TESS revealed similar patterns as STRUCTURE at K = 2 and 3 (data not shown), although *septentrionalis* was separated from the Central *sinicus* at K = 4 (Figures S4 and S5). The four clusters detected were generally concordant with the four mtDNA clades and four geographic groups (East, Central, Yunnan and Hainan), respectively (Figures 2c and d, S4 and S5).

### ABC Analysis

ABC analysis provided good support for the scenario of an ancient divergence between *septentrionalis* and *sinicus* with later divergence between Central *sinicus* and East/Hainan *sinicus* based on both DNA sequences and microsatellites datasets (scenario 3 in Figure S1, DNA sequences data, posterior probability = 1, the type I error rate is 0.25; microsatellite data, posterior probability = 1, the type error rate is 0.64).

### Estimates of Gene Flow

**Figure 6:**
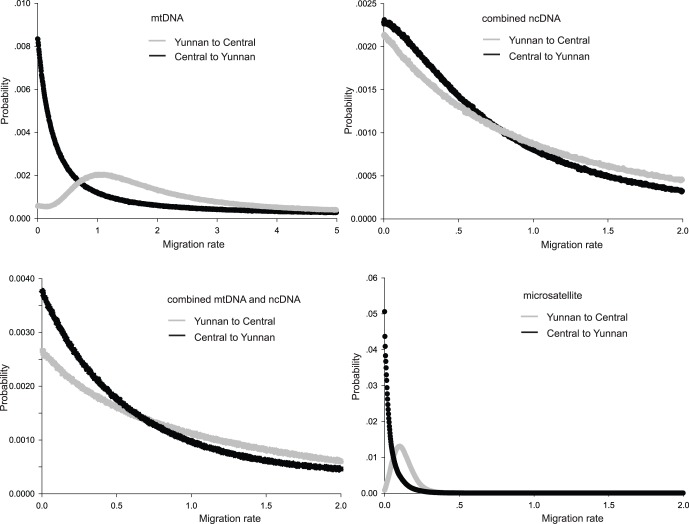
The marginal posterior probability distributions of migration rates between Central *sinicus* and *septentrionalis*. The IM analysis was performed for mtDNA, combined nuclear gene sequences, combined mtDNA and nuclear gene sequences and microsatellites, respectively.

Three independent IM analyses produced similar posterior distributions with effective sample sizes of >500 for all parameters, suggesting convergence on the true stationary distribution. Posterior probability distributions for migration rate based on four classes of datasets all had clear peaks and bounds within the prior distributions (Figure 6). The posterior mode and 90% credible intervals for migration rates are shown in Table 5. Estimates of gene flow using IM were markedly different among the four classes of markers used. Based on mtDNA, a moderate level of unidirectional gene flow was observed from *septentrionalis* (Yunnan) to the Central population of *sinicus*. In contrast, very restricted or no gene flow was detected between Yunnan and Central in either direction when IM analyses incorporated microsatellite data or ncDNA sequences, either alone or with mtDNA (see Table 5 and Figure 6).

**Table 5:** IM estimates of posterior mode and 90% credible intervals for directional migration rates (*m_1_* and *m_2_*).

Migration rate/markers	MtDNA	Combined ncDNA	Combined mtDNA and ncDNA	Microsatellite
Central vs YN				
*m_1_*	0.792	0.005	0.003	0.068
90% CI	0.298–1.478	0.001–1.497	0.001–1.661	0.008–0.162
*m_2_*	0.002	0.005	0.002	0.002
90% CI	0.001–0.414	0.001–1.603	0.001–0.818	0.001–0.131

## Discussion

### The Origin of *septentrionalis* and its Relationship with *sinicus*

Morphological, echolocation and nuclear DNA sequence data collected from *Rhinolophus sinicus* from across China supported the occurrence of the two subspecies *septentrionalis* and *sinicus*. Furthermore, phylogenetic results showed that these together form a monophyletic group and are thus true sister taxa although samples from its related taxa (e.g. *R.thomasi* and *R.rouxii*) will be needed to confirm this suggestion in the future. Therefore, of our three original hypotheses proposed to account for the origin and restricted range of *septentrionalis* nested within *sinicus*, we are able to reject the first scenario that the current taxonomy is incorrect, and so must consider the alternative explanations.

MtDNA gene trees and microsatellite-based clustering together revealed three divergent lineages within the subspecies *sinicus*, corresponding to Central China, East China and the offshore Hainan Island. However, contrary to morphological data, the Central lineage showed an unexpected closer relationship with *septentrionalis* than with other lineages of *sinicus.* The paraphyletic arrangement of *sinicus* with respect to *septentrionalis* could arise if, as stated in Hypothesis 2, the latter subspecies had recently evolved *in situ* from a Central lineage of its more widespread sister subspecies. However, phylogenetic discordance can also result from other processes such as incomplete lineage sorting of ancestral polymorphism or past mtDNA introgression [59], [60], which first need to be ruled out before accepting a recent origin of *septentrionalis*.

Several pieces of evidence in our study suggest that incomplete lineage sorting is an unlikely cause of the observed *sinicus* mtDNA paraphyly. First, the mtDNA haplotypes causing discordance were not distributed randomly across the range of *sinicus* as would be expected if this had been due to stochastic incomplete lineage sorting [2], but instead appeared in the parapatric regions of these two subspecies. Additionally, if incomplete lineage sorting had led to mtDNA paraphyly in *sinicus*, then we might expect to see similar patterns in the genealogies of nuclear genes given their larger effective population sizes [61], whereas we observed almost exclusive monophyly of each subspecies based on the markers *Chd1* and *SWS1* (Figure 4a and b). At the same time, however, such conflicts between mtDNA and ncDNA networks, plus the geographically localized nature of the discordant haplotypes, add considerable weight to the possibility of asymmetrical mtDNA introgression between female *septentrionalis* in Yunnan and male *sinicus* in the adjoining Central areas. This scenario also received support from the results of the IM analysis that indicated moderate mtDNA gene flow from *septentrionalis* to Central *sinicus* (Table 5). Similar cases of historical and contemporary introgression among related horseshoe bat taxa have been reported previously, and also appear to occur asymmetrically [12], [62].

For more formal tests of the hypotheses of a derived origin of *septentrionalis* versus introgression with *sinicus*, we applied coalescent-based phylogenetic reconstruction and Approximate Bayesian computation (ABC). From the former analysis, we found that contrary to expectations, the estimated species tree showed *septentrionalis* to be ancestral to *sinicus* (Figure 4e) and, therefore, a recent origin of this former subspecies (hypothesis 2) can be ruled out. It is noteworthy that *septentrionalis* was also found to show higher nuclear genetic diversity than regional populations of *sinicus* (Table 3), and was also characterized by a private yet frequent (28%) microsatellite allele. Such findings contradict predictions of a recent origin of *septentrionalis* but are consistent with a long evolutionary history of *septentrionalis*. ABC analysis also strongly supported the scenario of *septentrionalis* as the ancestor of all *R. sinicus* bats, although the type I error rate was high based on the microsatellite data. On the other hand, IM results offered further evidence of asymmetric mtDNA gene flow from *septentrionalis* into central populations of *sinicus* yet no nuclear gene flow, so again strongly pointing to historical mtDNA introgression.

### The History and Possible Causes of Lineage Divergence in *R. sinicus*

Accepting an ancient origin of *septentrionalis*, with subsequent mtDNA introgressive hybridisation with *sinicus*, raises questions about the likely forces that have led to the relative distributions of these taxa. Our results from mtDNA and microsatellites both revealed high genetic differentiation and strong phylogeographic structuring within *R. sinicus*, with four independent lineages resolved, i.e. *septentrionalis* and three *sinicus* lineages from Central, East and Hainan (see Figure 2c and d). Similar to the congeneric horseshoe bat *R. affinis* [12] in which the divergence dates between each mtDNA clade corresponded well to the period of glaciations in Pleistocene, strong phylogeographic differentiation within *R. sinicus* might result from the effects of Pleistocene climatic fluctuations. Specifically, TMRCA obtained for all *R. sinicus Cytb* sequences was estimated to be around 1.5 million years BP, while TMCRA estimates for *septentrionalis* and Central *sinicus* were similar (95% CI 140,000–500,000 years BP). This latter period coincides with the penultimate glacial period of Pleistocene [63] when numerous species will have undergone range contractions into multiple refugia where isolation could have promoted genetic divergence via drift [10]. Evidence for refugia in the east and southwestern plateaus of China has been reported by previous phylogeographic studies, e.g. in frogs [13] and in bats [14], [15]. In our study, a refugium in the southwest is indicated by high nuclear diversity observed in *septentrionalis,* and one in the East by both high mtDNA diversity and an ancient TMRCA estimate for bats in this region. Similarly, the Central area harbours high genetic diversity (mtDNA and ncDNA), shows morphological distinctiveness [64], and represents an independent lineage in phylogenetic analyses, so might have originated from yet another refugial area.

Given the distribution of *R. sinicus*, the observed strong genetic differentiation among lineages might have also been caused, at least in part, by geological and ecological differences between regions associated with the dramatic uplift of Qinghai-Tibetan Plateau (QTP) in the Quaternary [16]. This phenomenon had major consequences for latitudinal climatic clines between the east and the west of East Asia [16], and led to the formation of three marked altitudinal steps that also correspond to different ecological zones (see Figure 1 in ref. [15]). Relating this topography to our focal populations, *septentrionalis* corresponds to the edge of step 1, the Central *sinicus* population to step 2, and the East *sinicus* population to step 3. Because each period of uplift of the QTP had different effects on different regions of East Asia [65], divergent selection might have been a main driver of lineage divergence as well as a barrier to gene flow (see also [66]). Further detailed analysis of the environmental variables in different regions will be needed to test the possible occurrence of divergent selection [67], and work is needed to establish whether past distributions and population sizes also fit with these explanations.

## Supporting Information

Figure S1 **Graphic representation of the three scenarios for the history of lineage divergence used in the DIY ABC analysis.** Graphs show the logistic regression, showing the posterior probabilities of three tested scenarios. N_5_ corresponds to a bottleneck lasting a time of *db* generations.
(EPS)

Figure S2 **Maximum parsimony tree and network based on** ***Cytb*** **haplotypes including one sequence of** ***R. thomasi*** **.** For the tree, node support is indicated with MP, NJ and ML bootstrap values and Bayesian posterior probabilities (given above and below the branch, respectively). For the network, each circle represents a single haplotype and the circle size is scaled by the haplotype frequency. Filled black circles represent missing or unsampled haplotypes. One unfilled circle represents haplotype of *R. thomasi*. The numbers in the network represent mutational steps between haplotypes.
(EPS)

Figure S3 **The plots of the log-likelihood value L(K) (a) and delta K value (b) based on ten runs for each values of K from 2 to 8.**
(EPS)

Figure S4 **Geographic distributions of genetic clusters identified by GENELAND at K = 4.**
(EPS)

Figure S5 **Geographic distributions of genetic clusters identified by TESS at K = 4.**
(EPS)
